# Data on the application of the focused beam reflectance measurement (FBRM): A process parameters dataset for the ethyl cellulose (EC) microparticles preparation by the solvent evaporation method

**DOI:** 10.1016/j.dib.2020.105574

**Published:** 2020-04-21

**Authors:** Muhaimin Muhaimin, Roland Bodmeier

**Affiliations:** a University of Jambi; b Freie Universitaet Berlin

**Keywords:** Ethyl cellulose, microparticle, solvent evaporation method, FBRM

## Abstract

The data article refers to the paper “Effect of solvent type on preparation of ethyl cellulose microparticles by solvent evaporation method with double emulsion system using focused beam reflectance measurement” [1]. Data presented here include the effect of solvent type, method of emulsification (an oil-in-water (O/W) or a water-in-oil-in-water (W/O/W)), external aqueous phase volume, stirring speed and ethyl cellulose concentration on the preparation of ethyl cellulose microparticles. Data also refer to the effect of above mentioned factors on the solidification rate, hardening time, particle size, particle size mean, chord length distribution (CLD) and chord count of microparticles. Additionally, data exhibit process parameters when emulsion droplets transformed into solid microparticles during fabrication. The transformation of the emulsion droplets into solid microparticles occured within the first 10, 10.5, 12 and 60 minutes (O/W), and the first 12, 11.5, 10 and 90 minutes (W/O/W) when dichloromethane/methanol (1:1), dichloromethane, ethyl acetate and chloroform were used respectively. Either in O/W and W/O/W emulsion system, Chloroform gave smallest square weighted mean chord length. In contrast, its chord counts was not to be the highest.

Specifications table

**Table:** 

Subject	Chemistry
Specific subject area	Polymer Chemistry
Type of data	Table
	Graph
	Figure
How data were acquired	FBRM, SEM
Data format	Raw
	Analyzed
Parameters for data collection	- To measure solidification time, data were collected from emulsion droplet which in time showed constant particle size which is assumed as formation of solid microparticles.
	- At the time when solid microparticles were formed, the FBRM will count particle size means as well as the distribution of particle size of those solid microparticles.
Description of data collection	When a particle is crossed by the beam of FBRM, its reflection will be detected as a chord. The length of solid microparticles were collected as the particle size data, while amount of particles distributed and detected by beam was recorded as particle count as well as the distribution of particle size.
Data source location	Berlin, Freie Universitaet Berlin, Germany
Data accessibility	My raw data is provided 'With this article'
Related research article	Muhaimin, Bodmeier, R., 2017, Effect of solvent type on preparation of ethyl cellulose microparticles by solvent evaporation method with double emulsion system using focused beam reflectance measurement, Polymer International, Vol. 66 (11), 1448-1455. https://doi.org/10.1002/pi.5436

## Value of the data

• The data can be used as reference in determining the solvent to be used in microparticles fabrication
• The data can be used as reference in determining which method of emulsification to be employed in fabrication of microparticles
• The data proved that FBRM can be used for on line monitoring of microparticle using solvent evaporation method
• The data can be used for further study on parameters which affect microparticles fabrication by solvent evaporation method when FBRM is used as on line monitoring tools

## 1 Data Description

**Fig. 1:**
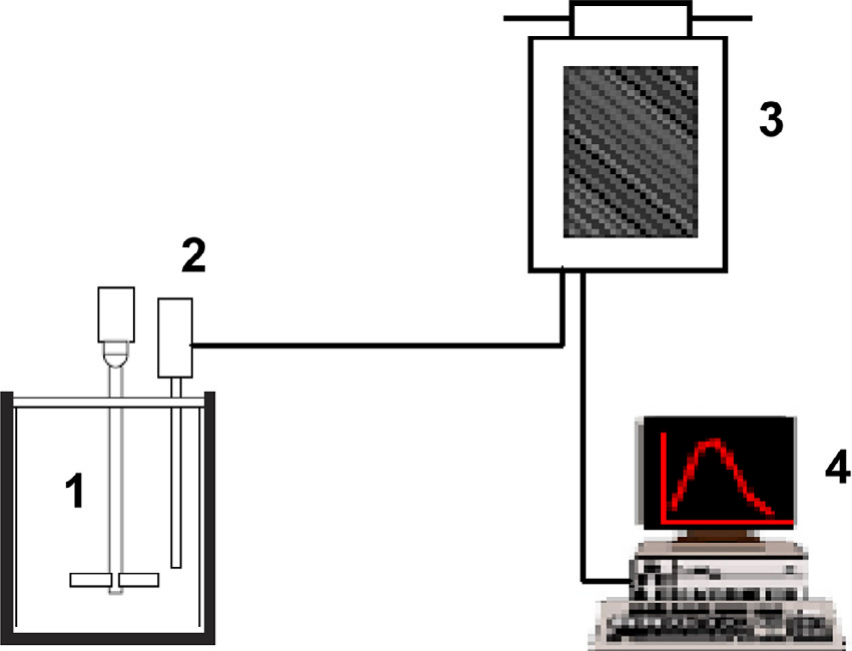
Schematic drawing of probe positioning relative to the impeller (1. Propeller stirrer; 2. Lasentec® FBRM probe; 3. Processing unit; 4. PC monitoring the particle size distribution on-line).

**Fig. 2:**
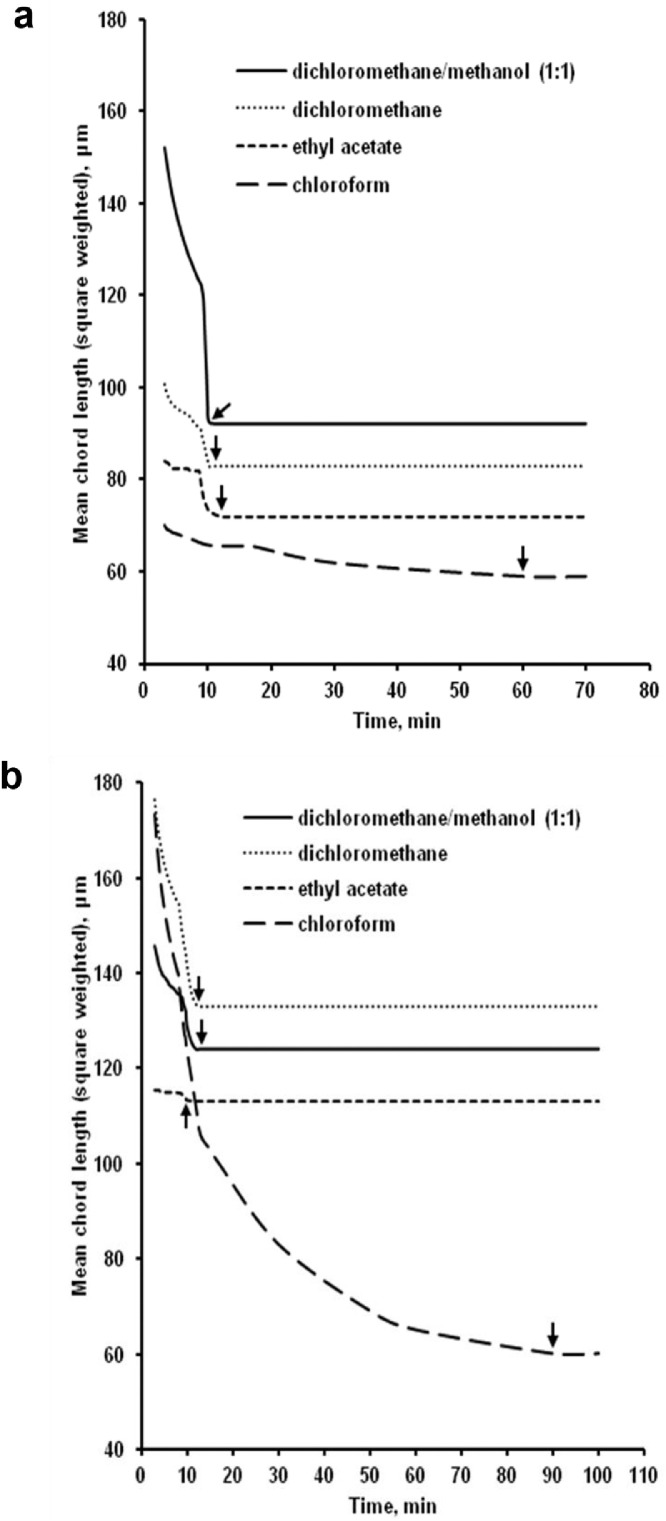
Effect of solvent type on square weighted mean chord length during microparticles formation by O/W (a) and W/O/W (b) [arrow (↓): starting time of microparticle hardening].

**Fig. 3:**
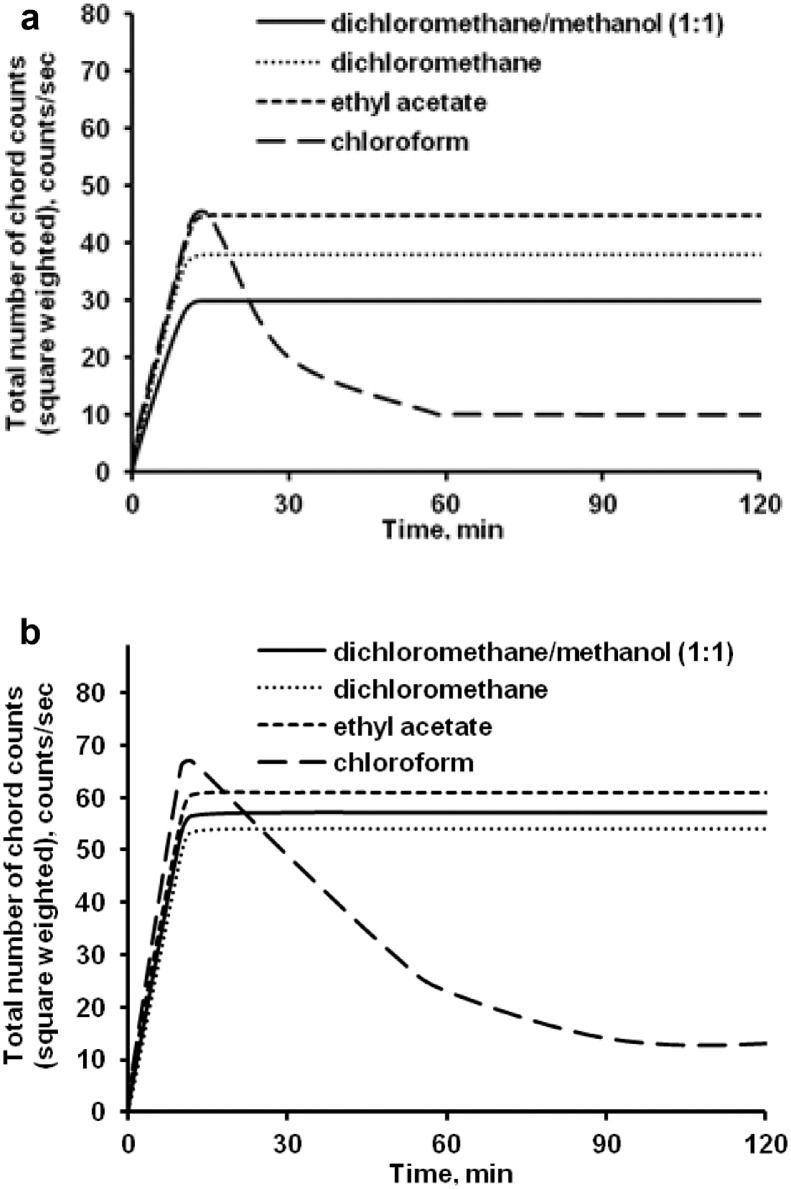
Effect of solvent type on the number of chord counts (square weighted) during solvent evaporation process [(a) O/W and (b) W/O/W method].

**Fig. 4:**
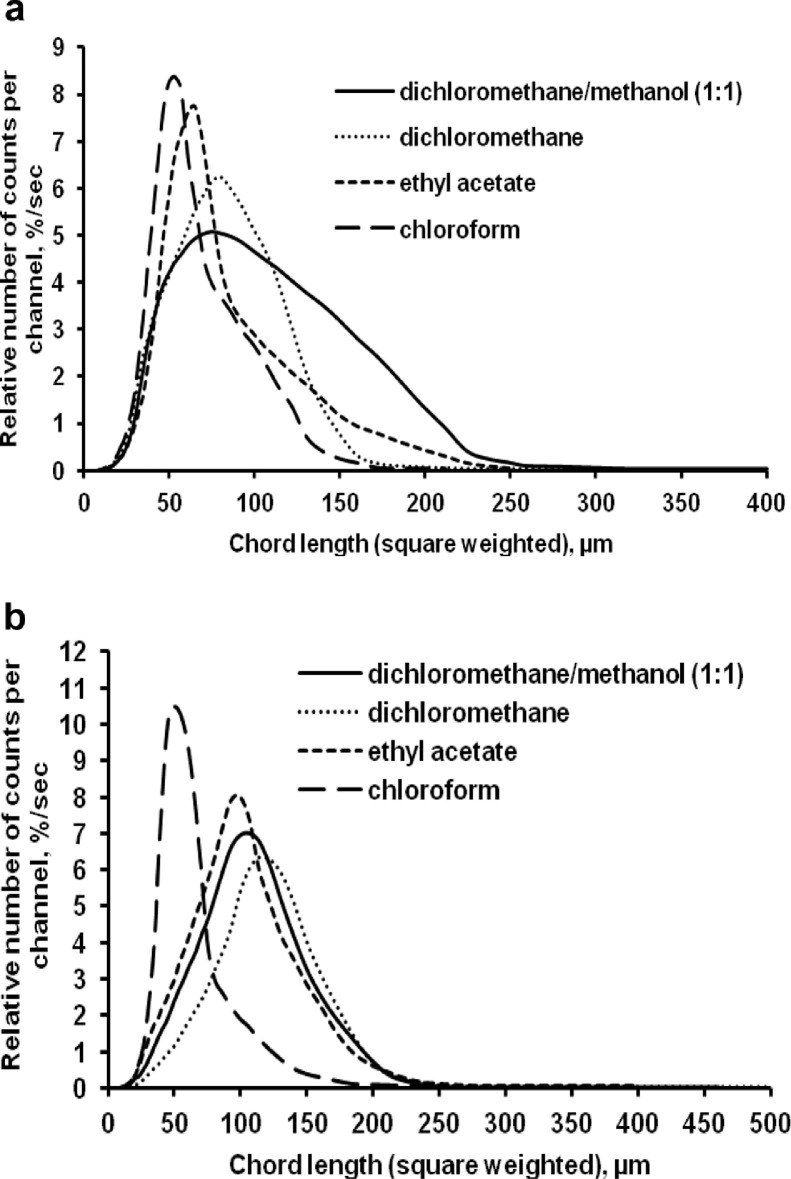
Effect of solvent type on the square weighted chord length distributions [(a) O/W and (b) W/O/W method, at 4 hours stirring time].

**Fig. 5:**
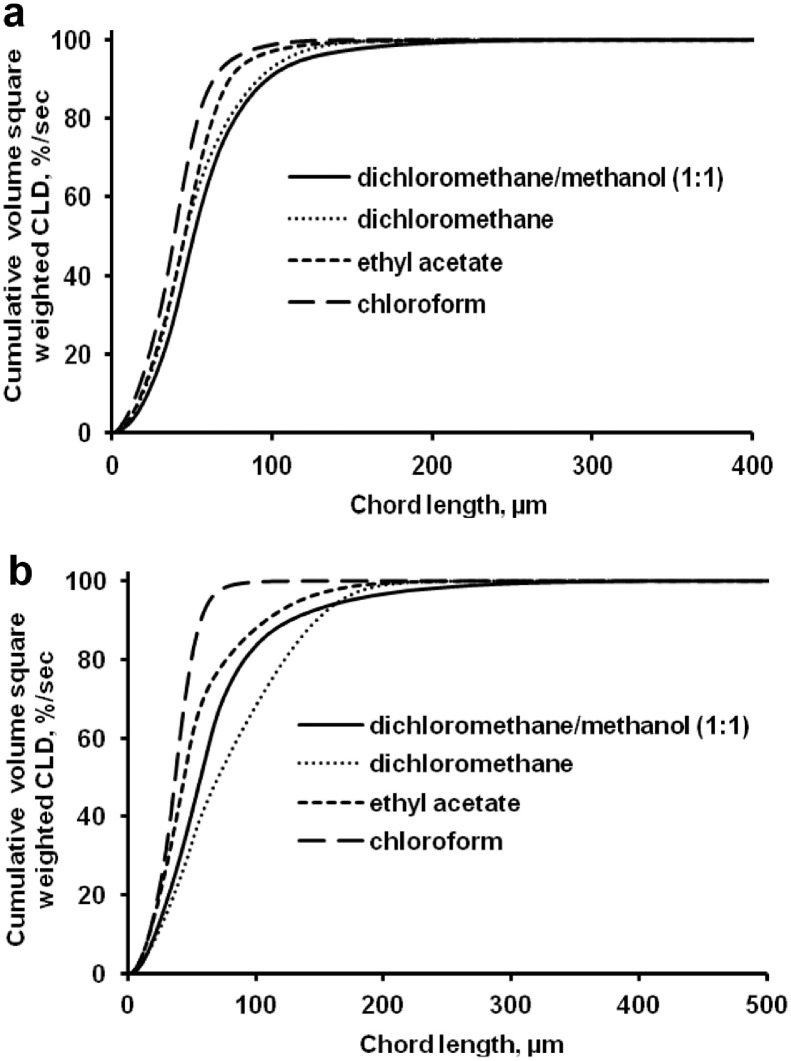
Comparison of the square weighted chord length distribution for various solvent obtained by the FBRM method [(a) O/W and (b) W/O/W; at 4 hours stirring time].

**Fig. 6:**
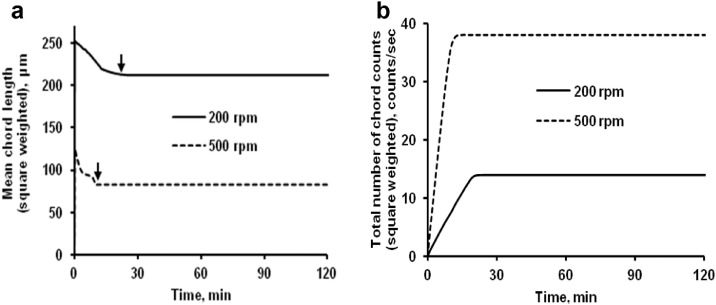
Effect of stirring speed on (a) square weighted mean chord length during microparticles formation and on (b) the number of chord counts (square weighted) during solvent evaporation process. (Arrow (↓): starting time of microparticle hardening).

**Fig. 7:**
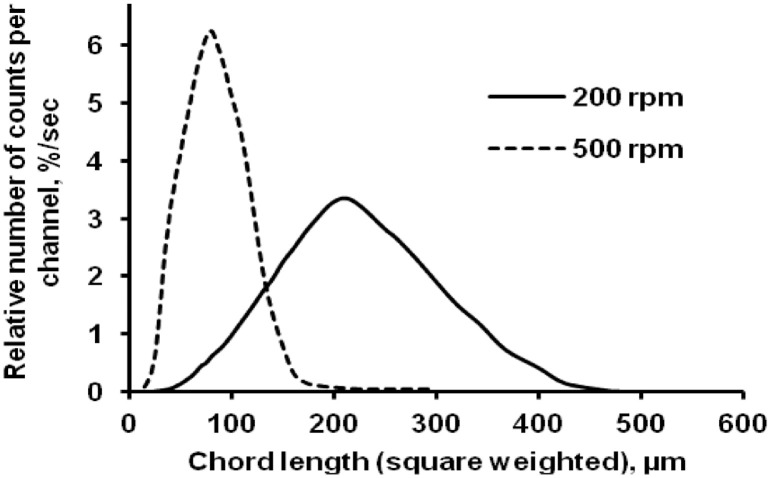
Effect of stirring speed on the square weighted chord length distributions (at 4 hours stirring time).

**Fig. 8:**
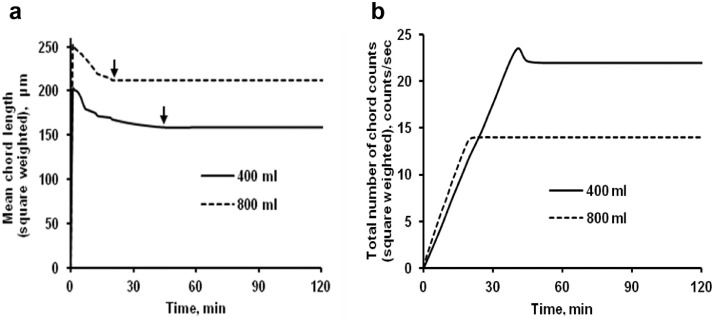
Effect of volume of external aqueous phase on (a) square weighted mean chord length during microparticles formation and on (b) the number of chord counts (square weighted) during solvent evaporation process. (Arrow (↓): starting time of microparticle hardening).

**Fig. 9:**
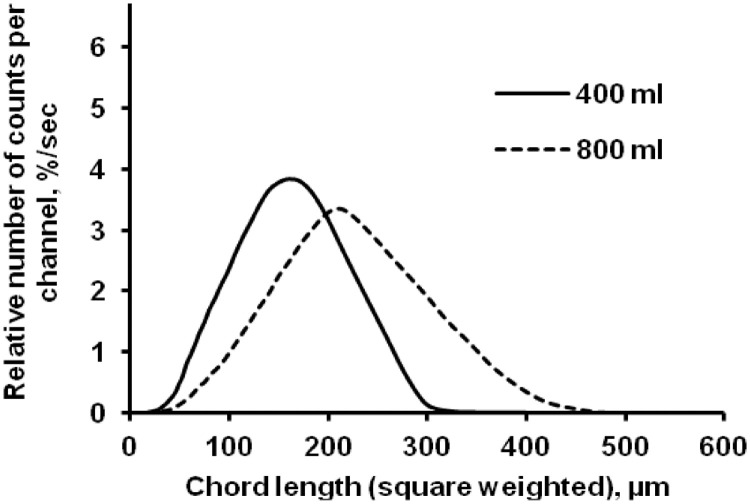
Effect of volume of external aqueous phase on the square weighted chord length distributions (at 4 hours stirring time).

**Fig. 10:**
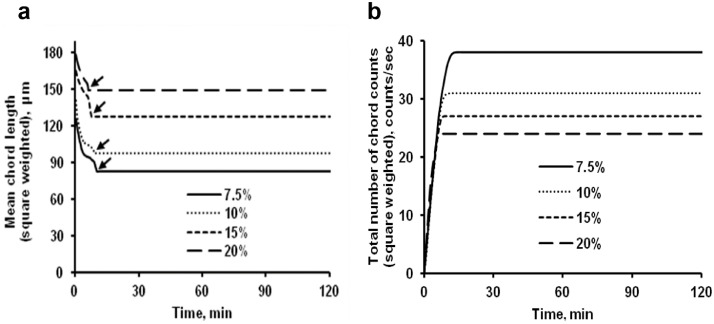
Effect of polymer concentration on (a) square weighted mean chord length during microparticles formation and on (b) the number of chord counts (square weighted) during solvent evaporation process. (Arrow (↓): starting time of microparticle hardening).

**Fig. 11:**
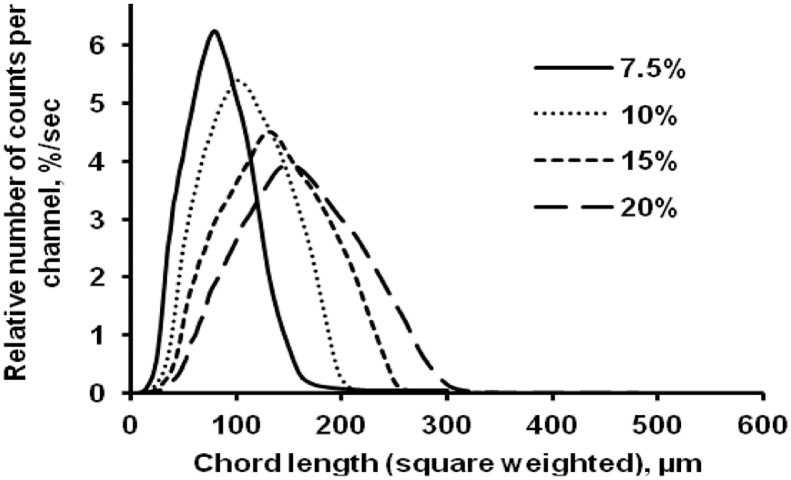
Effect of polymer concentration on the square weighted chord length distributions (at 4 hours stirring time).

**Fig. 12:**
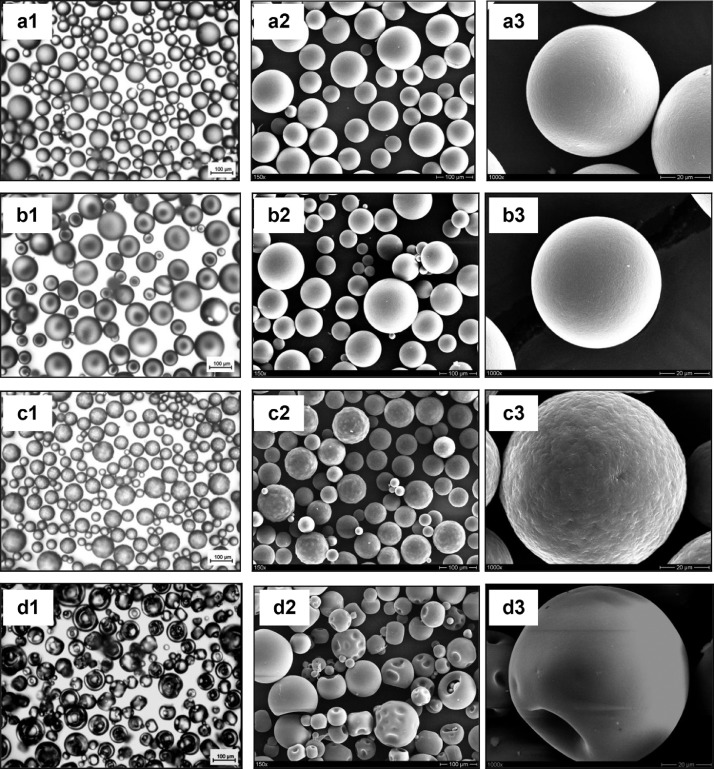
Optical microscopy pictures (1) and SEM Photomicrographs (2. lower magnification; 3. higher magnification) of ethyl cellulose based microparticles which prepared by O/W (a. dichloromethane; b. dichloromethane:methanol (1:1); c. chloroform; d. ethyl acetate).

**Fig. 13:**
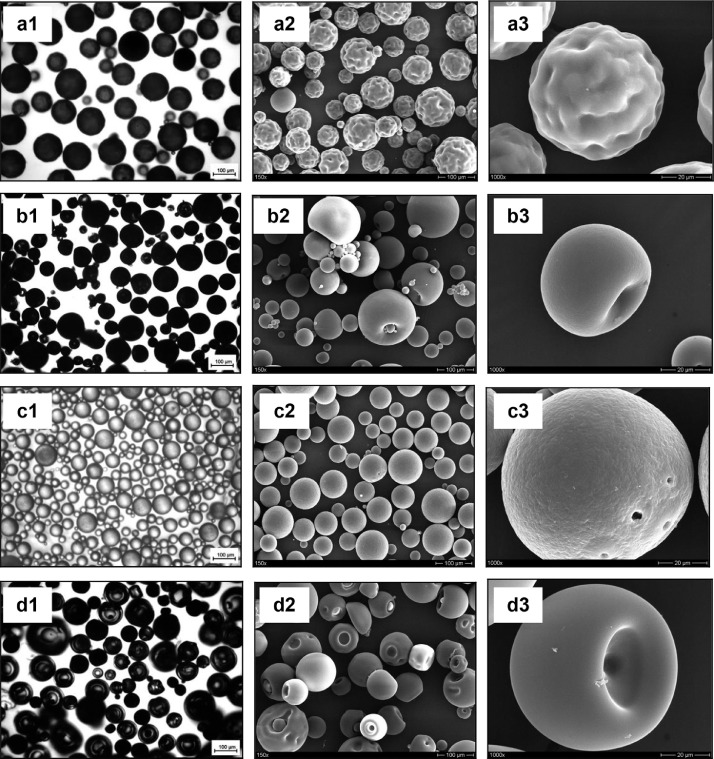
Optical microscopy pictures (1) and SEM Photomicrographs (2. lower magnification; 3. higher magnification) of ethyl cellulose based microparticles which prepared by W/O/W (a. dichloromethane; b. dichloromethane:methanol (1:1); c. chloroform; d. ethyl acetate).

**Fig. 14:**
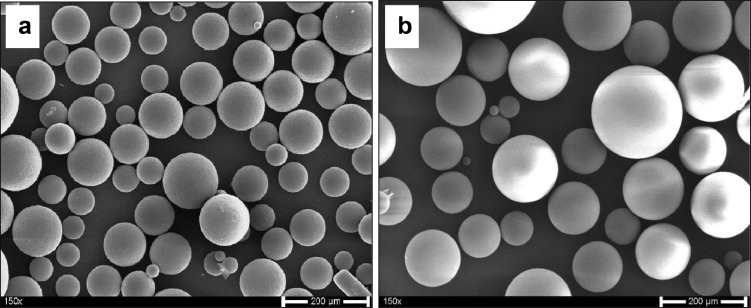
SEM photomicrographs of ethyl cellulose based microparticles with different polymer concentration. (a. 7.5% (w/v); b. 10% (w/v)).

The data presented here describe the influence of solvent type and emulsification method, on particle size of ethyl cellulose microparticles (Table 1). Tables 2 and 3 shows effect of those two parameters on the ethyl cellulose solubility, viscosity of ethyl cellulose solution, solidification rate (hardening time) and particle size mean of ethyl cellulose microparticles. On line monitoring on the effect of solvent type on square weighted mean chord length during microparticles formation at different method of emulsification (O/W and W/O/W) was performed by employing FBRM method (Fig. 1, Fig. 2) [1], [2], [3], [4], [5], [6], [7]. Effect of solvent type on the number of chord counts (square weighted) during evaporation process when using different method of emulsification was shown in Fig. 3a–b. The above mentioned effect on the distribution of square weighted chord length after 4 hours stirring time using different method of emulsification (O/W and W/O/W) can be seen in Fig. 4-b. FBRM method can also be applied in on monitoring the effect of different solvent on distribution of square weighted chord length during fabrication of microcapsule using the O/W and W/O/W method (Fig. 5a-b) [1,5]. O/W system consist of ethylcellulose in different type of solvent (O) and water containing 0,25% PVA (w/v) (W) while W/O/W system consist of water (W), of ethylcellulose in different type of solvent (O) and water containing 0,25% PVA (w/v) (W). As an addition, on line monitoring by FBRM can study the effect of stirring speed on (a) square weighted mean chord length and on (b) the number of chord counts during evaporation process (Fig. 6). The effect of stirring speed on distribution of square weighted chord length can also be monitored (Fig. 7). Data on the effect of external phase volume on square weighted mean chord length (a) and the number of chord counts (b) during solvent evaporation process was also presented (Fig. 8). Effect of volume of external phase on distribution of square weighted chord length was also studied by FBRM (Fig. 9). The effect of ethyl cellulose concentration on CLD, chord counts, square weighted mean chord length and hardening rate of microparticles from emulsion droplets presented in Fig. 10 while the distribution of hardening time from droplets to solid microparticles presented in Fig. 11. Moreover, data on the effect of solvent on the morphological surface of microparticles with different as O/W (Fig. 12) or as W/O/W system (Fig. 13) were also presented. Further, different polymer concentration effect on surface morphology of microparticles was shown in Fig. 14.

**Table 1:** Effect of solvent type and preparation method on particle size mean of microparticles using different method of emulsification.

Solvent	Particle size mean (µm) (± SD)			
	FBRM		Optical microscope	
	O/W	W/O/W	O/W	W/O/W
dichloromethane	83.24 (± 5.28)	133.28 (± 4.36)	88.78 (± 7.64)	137.51 (± 5.82)
dichloromethane/methanol (1:1)	92.17 (± 4.15)	124.41 (± 6.17)	95.06 (± 8.57)	129.64 (± 6.59)
chloroform	59.31 (± 6.47)	60.25 (± 5.35)	61.39 (± 5.18)	64.15 (± 3.95)
ethyl acetate	73.11 (± 5.13)	113.39 (± 3.19)	77.46 (± 8.25)	116.75 (± 7.35)

**Table 2:** Effect of solvent type and preparation method on solubility and viscosity of EC solution.

Solvent	Solubility (g/ml) (± SD)	Viscosity (cSt) (± SD)	
		O/W	W/O/W
dichloromethane	0.86 (± 0.03)	10.31 (± 1.14)	17.52 (± 1.25)
dichloromethane/methanol (1:1)	0.64 (± 0.06)	13.92 (± 1.03)	15.74 (± 1.17)
chloroform	1.06 (± 0.04)	9.08 (± 0.94)	9.71 (± 1.06)
ethyl acetate	0.79 (± 0.02)	11.45 (± 1.06)	14.85 (± 1.21)

**Table 3:** Effect of solvent type and preparation method on hardening time of microparticles.

Solvent	Hardening time (min)	
	O/W	W/O/W
dichloromethane	10.5	11.5
dichloromethane/methanol (1:1)	10	12
chloroform	60	90
ethyl acetate	12	10

## 2 Experimental Design, Materials, and Methods

### 2.1 Microparticle preparation

Microparticles were prepared by solvent evaporation methods using either W/O/W or O/W emulsion system [1,5]. In W/O/W emulsion, water was emulsified with solution of ethylcellulose in different solvent, to which dichloromethane, chloroform, ethyl acetate or dichloromethane:methanol (1:1) (7.5% w/v). Sonication was performed for 30 s (Sonoplus® HD 250, Bandelin Electronic GmbH & Co. KG, Berlin, Germany) under ice-cooling. This primary W/O emulsion was then dispersed into 800 ml 0.25% PVA (w/v) solution as external aqueous phase. Next, the emulsion was stirred for 4 h at 500 rpm using a propeller stirrer (Heidolph Elektro GmbH & Co. KG, Kelheim, Germany). Microparticles were collected by wet sieving and were washed with deionized water. The microparticles were dried at ambient temperature for 24 h and kept in a desiccator during storage.

In O/W-dispersions method, polymer Ethylcellulose (7.5% w/v) was dissolved in either dichloromethane, chloroform, ethyl acetate or dichloromethane:methanol (1:1) and then was dispersed into 0.25% PVA (w/v) solution (800 ml) as external aqueous phase. The emulsion was stirred by a propeller stirrer for 4 h at stirring speed of 500 rpm. The microparticles were collected and dried with similar process applied to W/O/W method.

Investigation on the effect of external aqueous phase volume were conducted by using a solution of Ethylcellulose (7.5% w/v) in dichloromethane using 400 ml and 800 ml water with 0.25% PVA (w/v) as stabilizer. The emulsion was stirred with a propeller stirrer for 4 h at 200 rpm. The next steps of microparticle collection and drying were similar with processes mentioned above.

### 2.2 Viscosity measurement

The viscosity were measured using an Ostwald viscometer type 50111/Ia by dissolving 7.5% w/v Ethylcellulose 4 cP in either dichloromethane, dichloromethane:methanol (1:1), chloroform or ethyl acetate as solvent. The instrument constant was K = 0.05152 mm^2^/s^2^ (Schott-Geräte GmbH, Hofheim, Germany) at 25 °C (n = 3). The viscosity of emulsion system as O/W or W/O/W were analysed as well. The viscosity were calculated as follows and listed in Table 2:

ν=K.t

ν: kinematic viscosity (mm^2^/s or cSt)

K: instrument constant (mm^2^/s^2^)

t: flow time (s)

### 2.3 Solubility determination

The solubility of ethyl cellulose 4 cP was determined in 1 ml of four different solvents (dichloromethane, dichloromethane:methanol (1:1), chloroform and ethyl acetate). To assure that the saturated solution has already obtained, samples were sealed and shaken at 37 °C and 75 rpm (horizontal shaker GFL 3033, Gesellschaft für Labortechnik GmbH, Burgwedel, Germany) for 24 h (n = 3). The saturated solution was filtered and the solvent was evaporated at room temperature. The mass of ethyl cellulose film was determined by an analytical balance (Analytical Balance Sartorius Research A200S, Sartorius GmbH, Göttingen, Germany). Result of ethyl cellulose solubility test shown in Table 2.

### 2.4 Online investigation by FBRM measurement

The probe of FBRM (Lasentec® FBRM D600T, Mettler Toledo AutoChem, Inc., Redmond, WA, USA) was positioned in the emulsification vessel to ensure good flow against the probe window. This condition hence allowed representative flow of microparticle as sample to be measured (Fig. 1) [1], [2], [3], [4], [5], [6], [7]. The FBRM D600T probe allow measurement in the range of 0.25 - 4000 μm. A propeller stirrer with speed of 500 rpm was employed beside the probe for 4 hours. Schematic positioning of the experiment shown in Fig. 1. The on line measurements were performed every 10 seconds, in triplicate within 4 hours. The data then were extracted and calculated through the iC FBRM® 4.0 software (Mettler Toledo AutoChem, Inc., Redmond, WA, USA).

Investigation of hardening time using FBRM showed in Table 3.

### 2.5 Microparticle characterization

#### 2.5.1 Optical microscopy

Morphology of microparticles were observed with an optical light microscope (Axiotrop 50, Carl Zeiss AG, Jena, Germany). The samples were spread on microscope slides, and the picture were captured with an image analysis system (INTEQ Informationstechnik GmbH, Berlin, Germany) consisting of a digital camera (type MC1) and the EasyMeasure® software (version 1.4.1).

#### 2.5.2 Scanning electron microscopic studies

Further, the morphology of microparticles were characterized by imaging analysis in scanning electron microscopy (SEM, S-4000, Hitachi High-Technologies Europe GmbH, Krefeld, Germany). The microparticle samples were fixed on a glass holder with double-sided tape. They were coated by an argon atmosphere with fine gold to a thickness of 8 nm (SCD 040, Bal-Tec GmbH, Witten, Germany) in a high-vacuum evaporator.

The effect of solvent type on the morphological surface of ethyl cellulose microparticles with different as O/W or as W/O/W system shown in Fig. 12, Fig. 13, Fig. 14.

## Appendix Supplementary material

Image, application 1
